# IUTA: a tool for effectively detecting differential isoform usage from RNA-Seq data

**DOI:** 10.1186/1471-2164-15-862

**Published:** 2014-10-06

**Authors:** Liang Niu, Weichun Huang, David M Umbach, Leping Li

**Affiliations:** Biostatistics Branch, National Institute of Environmental Health Sciences, Research Triangle Park, Kragujevac, NC 27709 USA

**Keywords:** Isoform usage, RNA-seq, Compositional data, Transcriptome profiling, Tissue, R package

## Abstract

**Background:**

Most genes in mammals generate several transcript isoforms that differ in stability and translational efficiency through alternative splicing. Such alternative splicing can be tissue- and developmental stage-specific, and such specificity is sometimes associated with disease. Thus, detecting differential isoform usage for a gene between tissues or cell lines/types (differences in the fraction of total expression of a gene represented by the expression of each of its isoforms) is potentially important for cell and developmental biology.

**Results:**

We present a new method IUTA that is designed to test each gene in the genome for differential isoform usage between two groups of samples. IUTA also estimates isoform usage for each gene in each sample as well as averaged across samples within each group. IUTA is the first method to formulate the testing problem as testing for equal means of two probability distributions under the Aitchison geometry, which is widely recognized as the most appropriate geometry for compositional data (vectors that contain the relative amount of each component comprising the whole). Evaluation using simulated data showed that IUTA was able to provide test results for many more genes than was Cuffdiff2 (version 2.2.0, released in Mar. 2014), and IUTA performed better than Cuffdiff2 for the limited number of genes that Cuffdiff2 did analyze. When applied to actual mouse RNA-Seq datasets from six tissues, IUTA identified 2,073 significant genes with clear patterns of differential isoform usage between a pair of tissues. IUTA is implemented as an R package and is available at http://www.niehs.nih.gov/research/resources/software/biostatistics/iuta/index.cfm.

**Conclusions:**

Both simulation and real-data results suggest that IUTA accurately detects differential isoform usage. We believe that our analysis of RNA-seq data from six mouse tissues represents the first comprehensive characterization of isoform usage in these tissues. IUTA will be a valuable resource for those who study the roles of alternative transcripts in cell development and disease.

**Electronic supplementary material:**

The online version of this article (doi:10.1186/1471-2164-15-862) contains supplementary material, which is available to authorized users.

## Background

Alternative splicing is widespread in higher eukaryotes as a way to increase cellular and functional complexity [1–6]. Most human genes are alternatively spliced [7–9], and most human alternative spicing is tissue-specific [9, 10]. Recently, the Encyclopedia of DNA Elements (ENCODE) project catalogued human transcripts genome-wide in two major cellular sub-compartments (nucleus and cytosol) for 15 cell lines [11], thus providing the most in-depth picture to date of the human transcriptome. One finding from that study is that about 10–13 isoforms are expressed per gene per cell line [11].

Growing evidence suggests that alternative splicing is important in cell functions. For example, it plays an important role in cell development [12–16]. Recently, Merkin et al. [8] studied tissue-specific splicing patterns in four mammals and one bird and identified many exons exhibiting highly conserved tissue-specific splicing patterns. Differential alternative splicing may be associated with diseases [17]; for example, overexpression of four-repeat tau mRNA isoforms has been reported in progressive supranuclear palsy [18]. Abnormally spliced mRNAs are also found in a high proportion of cancerous cells [19].

Traditionally, the expression level of individual isoforms is quantified one isoform at a time using a specific primer. Recent advances in next-generation sequencing technology [20, 21] allow genome-wide profiling of isoforms (transcripts). Although RNA-Seq conveniently provides a global view of the transcriptome at the gene level, deconvolution of the overall expression of a gene into the expression of its individual isoforms from sequence reads is not trivial because similar isoforms can generate identical sequence reads. Many of the tools that have recently been developed for this purpose detect differences in expression between two groups of samples for each individual isoform of a gene [22–27], rather than for all its isoforms simultaneously.

In this paper, we focus on detecting differential isoform usage: “differential” meaning differences between two groups of samples and “isoform usage” denoting the set of relative abundances (proportions of total gene expression) of all isoforms of a gene. Isoform usage so defined is assessed by a vector of fractions summing to one. Consequently, detecting differential isoform usage for a given gene between two groups of samples is a different problem from that mentioned earlier of detecting differential expression for any particular individual isoform. The former problem, by focusing on the entire compositional vector, implicitly incorporates the constraint that the fractional contributions of the component isoforms sum to one.

Existing methods for detecting differential isoform usage can be categorized into two types depending on whether or not they use information on isoform structure, e.g., gene annotation. Methods that require isoform structures, either known *a priori* or inferred from RNA-Seq data, include Cuffdiff2 [26], the chi-square test in [28], rDiff.parametric in [29] and the Probability Splice Graph (PSG) model in [30]. Methods that do not depend on isoform-structure information include the Flow Difference Metric (FDM) model in [31], DiffSplice in [32] and the rDiff.nonparametric in [29]. All of these methods essentially test for a difference between two groups in their underlying distributions of isoform usage; and they all make use of alignment data obtained from the RNA-Seq sequence reads (either single-end reads or paired-end reads).

Among methods that utilize prior information on isoform structure, Cuffdiff2 [26] either uses the known isoform-structure information or uses information on isoform structure inferred from the RNA-Seq alignment data by Cufflinks [33]. The alignment data are also used to estimate the abundance of isoforms of genes. These estimates are then used to test for differential isoform usage between the two groups for those genes with all isoforms sharing the same start site. Another method in this category, the chi-square test in [28], first utilizes the known isoform-structure information to identify regions that are unique to particular isoforms and uses the counts of the alignments in those unique regions to test for differential isoform usage. Similarly, for each gene, rDiff.parametric [29] first identifies genomic regions that are not common to all isoforms in the gene and uses the counts of the alignments in those regions to test for differential isoform usage by a negative-binomial model. Finally, PSG [34], uses known isoform structure information to construct a splice graph, aligns the RNA-Seq reads to the splice graph, estimates the weights of the edges in each sample from the aligned reads, then uses those estimated weights to test for differential isoform usage with a likelihood ratio test.

Each of these procedures has limitations, however. Cuffdiff2 cannot test for differential isoform usage directly when the isoforms of a gene do not share the same transcription start site (TSS), as it is designed to detect differential alternative splicing events for isoforms originating from the same pre-mRNA. The chi-square test in [28] can only be applied to genes that contain unique regions among the isoforms; so its power is expected to be limited when the unique regions are small. Similarly, rDiff.parametric [29] is expected to have limited power when regions that are not common to all isoforms are small. Finally, PSG [30] does not accommodate biological replicates and requires exactly one biological sample per group.

Tools that do not require isoform structures to test for differential isoform usage employ permutation tests to compare the alignments of sequence reads over the gene region in the two groups and provide an overall test for each gene. FDM [31] constructs a splice graph for each gene in each sample using the RNA-Seq alignment data, calculates the Flow Difference Metric (FDM, a metric to measure the difference between two above splice graphs) for every pair of the constructed splice graphs (one for a sample), and tests for differential isoform usage using those FDMs. DiffSplice [32] also constructs a splice graph from the RNA-Seq alignment data for each sample, and indirectly tests for differential isoform usage by testing for the differential distribution of the alignments over the genomic locations where “alternative splicing occurs” in the gene, i.e., the Alternative Splicing Modules (ASM’s) in [32]. rDiff.nonparametric represents each alignment in a gene as a binary vector whose length is the number of base pairs in the gene, calculates the average of these binary vectors from all samples in each group, and then tests for differential isoform usage using the Euclidean distance between the two averages as the test statistic. One advantage of these three procedures is that they do not require the isoform structure information and so can be applied to genomes with incomplete or unknown annotations. However, there are disadvantages too. For example, FDM and DiffSplice do not use the available data efficiently because, when applied to paired-end reads, they do not directly incorporate the uncertainty of the unsequenced portion of the underlying mRNA fragments into their models. Such uncertainty is only partially handled by alignment tools such as MapSplice (for FDM) and MapPER (for DiffSplice) before the data analysis. Also, none of these methods can pinpoint which isoforms are involved in differential isoform usage, as individual isoforms are never identified in these methods.

In this paper, we present Isoform Usage Two-step Analysis (IUTA), a novel approach for detecting genes with differential isoform usage between two groups of samples. IUTA requires knowledge of the gene’s isoform structure and availability of multiple samples (at least two per group). For a given gene, IUTA first estimates isoform usage in each sample based on paired-end RNA-Seq alignment data using a statistical model similar to one used by MISO [23]. IUTA then tests for differential isoform usage between the two groups using those estimates. Because isoform usage is a type of compositional data, i.e., vectors that contain the proportions of each component comprising the whole unit [35], IUTA defines the statistical testing problem as a test of equal means for two multivariate distributions under the Aitchison geometry [36] instead of under the usual Euclidean geometry. The Aitchison geometry is widely regarded more suitable for compositional data analysis than Euclidean geometry [37]. Fully elucidating the reasons this preference is beyond our scope here, but the following example will illustrate the issue. Consider two three-isoform genes in two conditions. For Gene A, isoform usage is (0.05, 0.55, 0.40) in Condition 1 and (0.10, 0.50, 0.40) in Condition 2. For Gene B, the corresponding isoform usage vectors are (0.30, 0.30, 0.40) and (0.35, 0.25, 0.40). Most biologists, regarding fold-change as the way to assess differential expression, intuitively regard Gene A has having a greater disparity in isoform usage between the conditions than Gene B because the proportion of the first isoform doubles for Gene A but only increases about 14% for Gene B and the changes in the second components are more comparable between the genes. The Euclidean distance between conditions is the same for both genes (the absolute change in first and second components is each 0.05); whereas the Aitchison distance between conditions is based on log-ratios (see Additional file 1) and is larger for Gene A compared to Gene B (0.609 vs. 0.238), in accord with intuition. IUTA is the first approach that tests for differential isoform usage under Aitchison geometry. IUTA is implemented as a R package and is available at http://www.niehs.nih.gov/research/resources/software/biostatistics/iuta/index.cfm.

We performed several simulation studies to show the performance of IUTA and to compare it with the Cuffdiff2 (version 2.2.0, released Mar. 2014), in which the developers improved the related testing procedures substantially from their old published procedures [26]. We restricted our comparison to Cuffdiff2, rather than including other methods that detect differential isoform usage, because Cuffdiff2 is perhaps the most commonly used tool that both tests for differential isoform usage and estimates isoform usage vectors. The results showed that IUTA is robust and has advantages over Cuffdiff2. We also applied IUTA to six tissue-specific RNA-Seq datasets from the Mouse Genomes Project at the Wellcome Trust Sanger Institute. We carried out 15 pair-wise comparisons among the six tissues to identify both genes with differential isoform usage between any two given tissues and genes with unique isoform usage in a given tissue compared to all others. This analysis represents the first genome-wide analysis of isoform usage in those tissues. Our results on those data are consistent with other biological literature and will be valuable to those who study tissue-specific isoform usage.

## Methods

### Overview of IUTA

Suppose we have paired-end RNA-Seq alignment data for multiple samples from each of two groups (at least two samples per group). As a first step, for each gene in each sample, IUTA uses alignment data (in BAM format) to estimate the isoform usage according to the isoform structure from an annotation file. IUTA employs a statistical framework that is similar to, but different in some aspects from MISO’s [23]. In a second step, IUTA uses the estimates from the first step to test for differential isoform usage in each gene between the two groups.

### Estimating isoform usage

Suppose a gene has *K* isoforms. Let ***θ***_*ij*_ = (*θ*_*ij*1_, ⋯, *θ*_*ijK*_) be the isoform usage of the gene for sample *j* in group *i* where *i* = 0, 1 and 1 ≤ *j* ≤ *J*_*i*_ (*J*_*i*_ is the number of samples in group *i*). Each *θ*_*ijk*_ represents the relative abundance of isoform *k*, that is, the proportion of the total number of transcripts for that gene with isoform *k*. To define a likelihood function for ***θ***_*ij*_, we let *l*_*k*_ be the length of isoform *k* (1 ≤ *k* ≤ *K*), and introduce the following notation, in which the group and sample indices *i* and *j* are ignored for the sake of brevity. Let ***r*** = (*r*_1_, ⋯, *r*_*N*_) be the observed alignment data over the gene region, where *N* is the number of aligned paired-end sequence reads and *r*_*n*_ (1 ≤ *n* ≤ *N*) is the alignment, i.e., the genomic location information for the pair *n*. Let ***R*** = (*R*_1_, ⋯, *R*_*N*_) be the random vector for which *r* is a realization. For *n* = 1, ⋯, *N*, let *I*_*n*_ be the random variable indicating the isoform from which read *R*_*n*_ is sequenced; let *F*_*n*_ be the random variable representing the fragment from which read *R*_*n*_ is sequenced; and let *L*_*n*_ be the random variable representing the length of *F*_*n*_. Also let *l*_*k*_^*n*^ be the length of the fragment of isoform *k* (1 ≤ *k* ≤ *K*) that matches *r*_*n*_ (1 ≤ *n* ≤ *N*), i.e., the two ends of the fragment are identical with read pair *n*, and define *l*_*k*_^*n*^ as 0 if there is no fragment of isoform *k* that matches *r*_*n*_. Let *f*( · )be the fragment length distribution (unknown but can be estimated from the data).

The likelihood function for a single gene (in sample *j* of group *i*) is then defined as:


Because *P*(*R*_*n*_ = *r*_*n*_, *I*_*n*_ = *k*|*θ*) = *P*(*R*_*n*_ = *r*_*n*_, *I*_*n*_ = *k*, *L*_*n*_ = *l*_*k*_^*n*^|*θ*), we can write


Expressing the joint probability as a product of conditional probabilities, we have


where *k* = 1, ⋯, *K*. The unknown fragment length distribution *f*(·) can be estimated from *r* (Additional file 1). Note that the sample index (*ij*) of *p*_*k*_ (should be *p*_*kij*_) is also ignored in the above discussion.

IUTA’s likelihood function is similar to the likelihood function in MISO [23], with three main differences. First, MISO defines *p*_*k*_ differently than IUTA does, using  where *μ* is the mean of the empirical fragment length distribution *f*’ that is obtained by mapping all paired-end reads to long constitutive 3′ UTRs (UnTranslated Regions). Second, MISO uses a discrete normal distribution (with mean and variance pre-determined by the empirical fragment length distribution in any RNA-Seq sample processed by the same assay) in the place of *f* while we estimate *f* from the analyzed RNA-Seq sample in a completely different way (Additional file 1). Finally, IUTA and MISO calculate *P*(*R*_*n*_ = *r*_*n*_|*I*_*n*_ = *k*, *L*_*n*_ = *l*_*k*_^*n*^, *θ*) differently: IUTA uses  while MISO uses , where *m*(*r*_*n*_, *l*_*k*_) is the number of “mappable positions” defined to reflect an overhang restriction when a read straddles a splice junction.

To find the maximum likelihood estimates (MLEs) of *θ*, we first use an expectation-maximization (EM) algorithm to find the MLE of *p*, denoted , based on the likelihood function *L*(*θ*) (treating the *I*_*n*_’s as unobserved latent variables). We then calculate the MLEs of ***θ***, denoted , from  where  (1 ≤ *k* ≤ *K*). The details of the EM algorithm, including the choice of the starting point of the algorithm, are provided in Additional file 1.

### Testing for differential isoform usage

Let *θ*_*i*_ be the mean isoform usage (in Aitchison geometry) of the gene of interest in group *i*, where *i* = 0, 1. We want to test:


Using an isometric log-ratio (ilr) transformation [38], a distance-preserving one-to-one mapping from the open simplex  with Aitchison geometry to the real space ℝ^*K* − 1^ with Euclidean geometry, we can test the equivalent hypotheses:


The ilr transformation allows us to honor the Aitchison geometry on the simplex when carrying out statistical calculations in familiar Euclidean space. Details of the Aitchison geometry and the ilr transformation are provided in Additional file 1.

We take a hierarchical approach to deriving a probability model for a sample-specific isoform usage estimate , the MLE of the sample-specific mean isoform usage (***θ***_*ij*_) in sample *j* of condition *i* (this MLE is obtained from the EM algorithm previously mentioned)*.* Let  denote the random variable for sample-specific estimated isoform usage, whose observed value is . Our hierarchical model specifies a first-stage distribution for  conditional on the sample-specific mean ***θ***_*ij*_ and a second-stage distribution for *ilr*(***θ***_*ij*_) conditional on the group-specific mean ***θ***_*i*_. This second stage of the hierarchy implicitly views the ***θ***_*ij*_ as random quantities that vary around a fixed group-specific mean ***θ***_*i*_.

More specifically, we assumed in the first stage of the hierarchy that the distribution of , after *ilr*- transformation, has a normal distribution on ℝ^*K* − 1^ [39] centered at *ilr*(*θ*_*ij*_) with variance-covariance matrix **Υ**_*ij*_, that is,
1

where  denotes the multivariate normal distribution in ℝ^*K* − 1^. We require that  lie in the open simplex, i.e., that all relative abundances are positive so that the ilr transformation is applicable. To satisfy the requirement, if an isoform’s relative abundance is estimated as zero in some, but not all, samples, IUTA replaces values estimated as zero with a small positive value (0.0001 by default; adjustable by the user). If an isoform’s relative abundance values are estimated as zero in all samples of both groups, then that isoform is excluded from consideration in the testing stage. The variance-covariance matrix **Υ**_*ij*_ can vary across samples in the same group because read coverage may differ among samples. For the second stage of the hierarchy, we assumed that each sample-specific mean ***θ***_*ij*_ for 1 ≤ *j* ≤ *J*_*i*_, after *ilr*- transformation, arises independently from a normal distribution on ℝ^*K* − 1^ centered at *ilr*(***θ***_*i*_) with variance-covariance matrix **Σ**_*i*_, that is,
2

Similar to the first stage, we require that ***θ***_*ij*_ lie in the open simplex. Also note that the model allows each condition to have a distinct variance-covariance matrix **Σ**_*i*_. Combining Equations (1) and (2), gives the marginal probability model for sample-specific estimated isoform usage :


We simplified this model somewhat in our testing procedures by proceeding as though **Υ**_*ij*_ = **Υ**_*i*_ for 1 ≤ *j* ≤ *J*_*i*_. Although this simplifying assumption is arbitrary, a simulation study showed that IUTA is robust to moderate departures from it (see Additional file 1). Consequently, the statistical tests implemented in IUTA assume that all samples within group *i* have a common marginal variance-covariance matrix, **Σ**_*i*_ + **Υ**_*i*_, that differs between groups.

Thus, under our assumptions, testing *H*_0_’ versus *H*_1_’ is a test of whether two multivariate normal distributions have equal mean vectors when they have different variance-covariance matrices. In the statistical literature, this problem is called the multivariate Behrens-Fisher problem. We employed the SKK [40], CQ [41] and KY test [42] to test for *H*_0_’ versus *H*_1_’. Details are provided in Additional file 1. Notice that when *K* = 2, the KY test becomes the Welch’s t-test [43]. In implementing these tests, IUTA employs the observed values of  from each group to empirically estimate the group-specific variance-covariance matrix **Σ**_*i*_ + **Υ**_*i*_ for each group *i*. The KY test requires that the estimate of **Σ**_*i*_ + **Υ**_*i*_ be positive definite for each group; but the SKK and CQ tests do not, though their critical values are justified using large-sample theory. These latter two tests are appropriate for situations where the number of isoforms in a group is greater than the number of samples in the group. Consequently, IUTA can in principle accommodate genes with any number of isoforms provided there are at least two samples per group (though statistical power would, of course, suffer with such small sample sizes).

### Simulations

We performed three simulation studies. The first one aimed at two goals: 1) to compare the three tests (SKK, CQ and KY) implemented in IUTA, and 2) to compare IUTA (with SKK) with Cuffdiff2 (version 2.2.0, release in Mar. 2014). The second simulation studied the robustness of IUTA to the violation of the constant variance-covariance assumption that **Υ**_*ij*_ = **Υ**_*i*_ for 1 ≤ *j* ≤ *J*_*i*_. We induced differences in **Υ**_*ij*_ among samples by simulating differences in read coverage among samples. The third aimed to assess further the robustness of IUTA to differences of read coverage among samples.

In the first two simulation studies, we selected 8,628 mouse genes with number of isoforms between two and ten from the UCSC known mouse genes (mm10) annotation [44]. In the last simulation study, we randomly selected five genes (*Zfp407*, *Loxl2*, *Bptf*, *Pde4dip*, and *Stab2*) with 2, 3, 5, 7 and 8 isoforms, respectively, and then two additional genes (*Dido1* and *Ifi203*), both with 8 isoforms. Details of gene selection are provided in Additional file 1.

In the first two simulation studies, we divided the 8,628 genes into two sets (8,060 genes with 2–5 isoforms and 568 genes with 6–10 isoforms). Using each set of genes, we simulated 10 RNA-Seq alignment datasets (five for each group) in BAM format (http://genome.ucsc.edu/FAQ/FAQformat.html#format5.1). The average read coverage for each gene in the first simulation study was 100, and the average read coverage for each gene in the second simulation study varied among the samples (either 30, 50, 70, 90, or 110) in each group. In the last simulation study, we chose six different levels of read coverage (10, 30, 50, 70, 90, and 110). At each read-coverage level, we carried out 1,000 *in silico* experiments for each gene. In each of the 1000 experiments, we also simulated 10 RNA-Seq alignment datasets (five for each group).

The parameters **Σ**_0_ and **Σ**_1_ used in the simulation studies for the 8,628 genes were obtained from Cufflinks analysis of 10 mouse placenta RNA-Seq data sets (five wild-type and five *Zfp36l3* knockout) (unpublished) provided by Perry Blackshear (NIEHS). The details of all simulations are provided in Additional file 1.

## Results

### Comparison among three different tests in IUTA

Our simulation study demonstrated that the SKK test and CQ test performed comparably and both outperformed the KY test (Additional file 1: Figure S1). As the KY test is only applicable when the dimension of the data is less than the sample sizes, we used the SKK test for all subsequent analyses.

### Performance comparison between IUTA and Cuffdiff2

**Figure 1:**
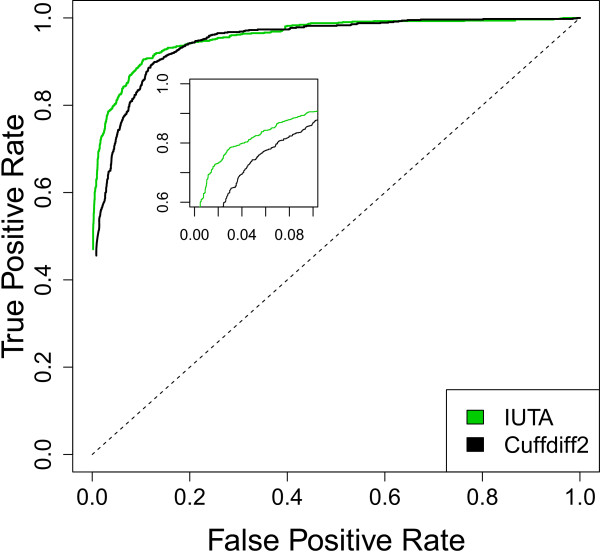
**Performance comparison between IUTA and Cuffdiff2 using simulated data.** Receiver Operating Characteristic (ROC) curves based on the 4159 mouse genes that can be tested by both Cuffdiff2 and IUTA. False positive rate (proportion of true negatives that are claimed as positives) and true positive rates (proportion of true positives that are claimed as positives) were computed by varying the p-value cutoffs.

We also used the above simulated datasets to compare the performance between IUTA and Cuffdiff2. Out of the 8,628 (see Additional file 1 for details on the gene selection) mouse mm10 UCSC known genes [44], IUTA was able to analyze all of them for differential isoform usage. In comparison, Cuffdiff2 could only analyze 4159 (48%). Cuffdiff2 was unable to analyze the other 4,469 genes for two reasons. First and foremost, Cuffdiff2 is designed specifically to test differential isoform usage for a given transcription start site (TSS). Nearly half of the genes (4,381 out of 8,628) contained isoforms with different TSSs, thus, Cuffdiff2 eliminated those genes from testing. Second, Cuffdiff2 is not well suited for testing genes whose isoform structures are similar. Even with the average reads coverage of 100 used in our simulation, Cuffdiff2 declared that 88 of the 4,247 did not have enough alignments for testing. For the 4,159 genes that both IUTA and Cuffdiff2 analyzed, IUTA outperformed Cuffdiff2 (Figure 1).

### Robustness of IUTA to a constant variance-covariance assumption

To see how IUTA performed when the constant variance-covariance assumption that **Υ**_*ij*_ = **Υ**_*i*_ for 1 ≤ *j* ≤ *J*_*i*_ (see the Methods section) is violated, we repeated the same simulations as above but varied the read coverage among the five samples of each group for each gene (either 30, 50, 70, 90, or 110), as this variation in coverage would induce variation in the precision with which isoform usage was estimated in each sample. The ROC curves for IUTA (Additional file 1: Figure S2) were nearly identical to those in Figure 1. Again, the SKK and CQ tests performed comparably and both outperformed the KY test. With smaller coverage per sample than in the previous simulation, Cuffdiff2 could analyze only 4,136 of the 8,628 genes that IUTA analyzed. As before, for the genes both tools could analyze, IUTA performed slightly better than Cuffdiff2. Those results demonstrated that IUTA is robust to the violation of the constant variance-covariance assumption.

### Robustness of IUTA to the depth of read coverage

For seven genes (*Zfp407, Loxl2, Bptf, Pde4dip, Stab2, Dido1* and *Ifi203*), we chose six different levels of average read coverage, namely, 10, 30, 50, 70, 90, and 110, and carried out 1,000 simulations at each read-coverage level. We ran IUTA on the 42,000 datasets (7 × 6 × 1000). Type-I error rate and power were computed based on the nominal α level 0.05. As shown in Table 1, the Type-I error rate and power were consistent across the different levels of read coverage for each gene, except for *Bptf*, a gene with five isoforms, two of which (uc007mac.1 and uc007mae.2) contain 28 long exons and are highly similar. Among the three genes (*Dido1*, *Ifi203*, and *Stab2*) with eight isoforms, IUTA had the smallest power for *Stab2*, a long gene (166,733 bp) with 69 exons for which the eight isoforms have many exons in common. For the other two genes (*Ifi203* and *Dido1*) with eight isoforms but with fewer exons in common, IUTA showed higher power. Therefore, we concluded that the read coverage had little effect the performance, but the isoform structure mattered.

**Table 1:** **Empirical Type I error rate (empirical power) of IUTA_SKK at nominal Type I error rate 0.05 for various levels of read coverage for a selection of genes**

Gene	No. of isoforms	Read coverage					
		10	30	50	70	90	110
*Zfp407*	2	0.06 (0.99)	0.08 (1.00)	0.08 (1.00)	0.08 (1.00)	0.08 (1.00)	0.07 (1.00)
*Loxl2*	3	0.07 (1.00)	0.07 (1.00)	0.10 (0.99)	0.09 (1.00)	0.11 (0.99)	0.11 (0.99)
*Bptf*	5	0.30 (0.96)	0.29 (0.96)	0.27 (0.96)	0.27 (0.95)	0.25 (0.95)	0.23 (0.96)
*Pde4dip*	7	0.04 (0.92)	0.04 (0.94)	0.07 (0.95)	0.04 (0.93)	0.06 (0.96)	0.07 (0.96)
*Dido1*	8	0.11 (0.87)	0.10 (0.89)	0.10 (0.90)	0.11 (0.88)	0.11 (0.87)	0.10 (0.89)
*Ifi203*	8	0.09 (0.77)	0.10 (0.76)	0.08 (0.77)	0.09 (0.70)	0.10 (0.76)	0.09 (0.74)
*Stab2*	8	0.08 (0.35)	0.07 (0.43)	0.06 (0.45)	0.07 (0.44)	0.07 (0.51)	0.08 (0.44)

Inspection of Table 1 revealed a troubling feature of IUTA_SKK: although the tests were performed at nominal α level 0.05, the empirical type I error rate (false positive rate) was larger than 0.05 for almost every simulated data set. IUTA_CQ, but not IUTA_KY, showed the same failure to maintain the correct Type I error rate (results not shown). Although we had not included CuffDiff2 in this simulation originally, we now undertook a similar simulation study to compare IUTA and CuffDiff2 with respect to Type I error rates. We selected three genes (*Dhx9*, *Mr1* and *Trim11*), each had 4 isoforms sharing the same transcript start site. We considered two different levels of read coverage (30 and 90). For each gene at each read-coverage, we simulated 1000 data sets with 5 replicates in each of the two groups under the null hypothesis that both groups had the same isoform usage. The empirical type I error rates for all three tests (IUTA_SKK, IUTA_CQ and Cuffdiff2) were larger than nominal 0.05 (Table 2). We conclude that both Cuffdiff2 and IUTA fail to control the Type I error rate at the nominal level.

**Table 2:** **Empirical Type I error rates at nominal Type I error rate 0.05 for three tests at two levels of read-coverage for three genes**

Gene	Read coverage	Test		
		IUTA_SKK	IUTA_CQ	Cuffdiff2
*Dhx9*	30	0.10	0.10	0.08
	90	0.10	0.10	0.09
*Mr1*	30	0.22	0.13	0.28
	90	0.25	0.13	0.28
*Trim11*	30	0.13	0.11	0.10
	90	0.12	0.11	0.11

Taken together, our simulation studies demonstrated that IUTA performed better than Cuffdiff2 for genes that both could analyze and was able to analyze more genes than Cuffdiff2. IUTA was robust to the read coverage, but both IUTA and Cuffdiff2 failed to maintain the nominal Type I error rate. IUTA was more powerful for genes whose isoforms have fewer exons in common than for genes with many exons in common.

### Applications of both IUTA and Cuffdiff2 to real RNA-Seq datasets from mouse tissues

We applied IUTA to 36 RNA-Seq datasets from the Mouse Genomes Project at the Wellcome Trust Sanger Institute (accession number: ERP000591). These datasets consist of six biological replicates from each of six mouse tissues (liver, spleen, lung, heart, thymus, and hippocampus).

We first aligned the sequence reads to the mouse (GRCm38 or mm10) RefGene gene models using Tophat (version 2.0.8) [45]. Next, we carried out all 15 pair-wise comparisons between any two tissues using IUTA. In each comparison, we estimated the isoform usage in each sample, gene by gene, and then tested for differential isoform usage while controlling the family-wise error rate (FWER) at 0.01 with the Bonferroni correction. The numbers of genes with statistically significant differential isoform usage are listed in Table 3 with their official gene symbols provided in Additional file 2. The smallest number of genes with differential isoform usage was from the comparison between spleen and heart whereas the highest number was from the comparison between thymus and hippocampus. In total, IUTA identified 2,073 genes with differential isoform usage in at least one comparison. Estimated isoform usage in all six tissues and the pair-wise comparison results (p-values) for all genes tested are provided in Additional file 3.

**Table 3:** **The number of genes with statistically significant differential isoform usage between pairs of mouse tissues**

Tissue	Liver	Spleen	Thymus	Lung	Heart	Hippocampus
Liver	NA	323	680	508	399	847
Spleen	323	NA	311	365	284	676
Thymus	680	311	NA	538	474	1030
Lung	508	365	538	NA	303	781
Heart	399	284	474	303	NA	459
Hippocampus	847	676	1030	781	459	NA

Next, we searched for genes with tissue specific isoform usage. Specifically, we searched for genes whose isoform usage was significantly different in all comparisons between a given tissue and remaining five tissues but non-significant in every comparison that did not involve the given tissue. Forty-six such genes were identified (Table 4).

**Table 4:** **Genes with significant tissue-specific isoform usages from 15 pair-wise comparisons among six tissues**

Tissue	Gene
Hippocampus	*Abi2, Arfgap2, Camta2, Dctn1, Dctn2, Eef1d, Eif4g1, Ltbp4, Nelf, Nqo2, Pxk, Tecr, Mon2, Traf3, Faim, Parp6, Gopc, Eya3, 1110021J02Rik, Nt5c2, Cadm1, Camkk2, Fchsd2, Gnal, Ptprs, Wdr37, Morf4l2*
Heart	*Naca*
Liver	*Mxi1, Psen2, Ptpn6, Bdh1, Nmi, Tomm6, Efemp2, Ythdf3, Slc6a8, Masp2, Il33, Stat5b, Timm22, Alkbh7*
Lung	*Cttnbp2nl, C1qtnf5*
Spleen	*Fam149b*
Thymus	

**Figure 2:**
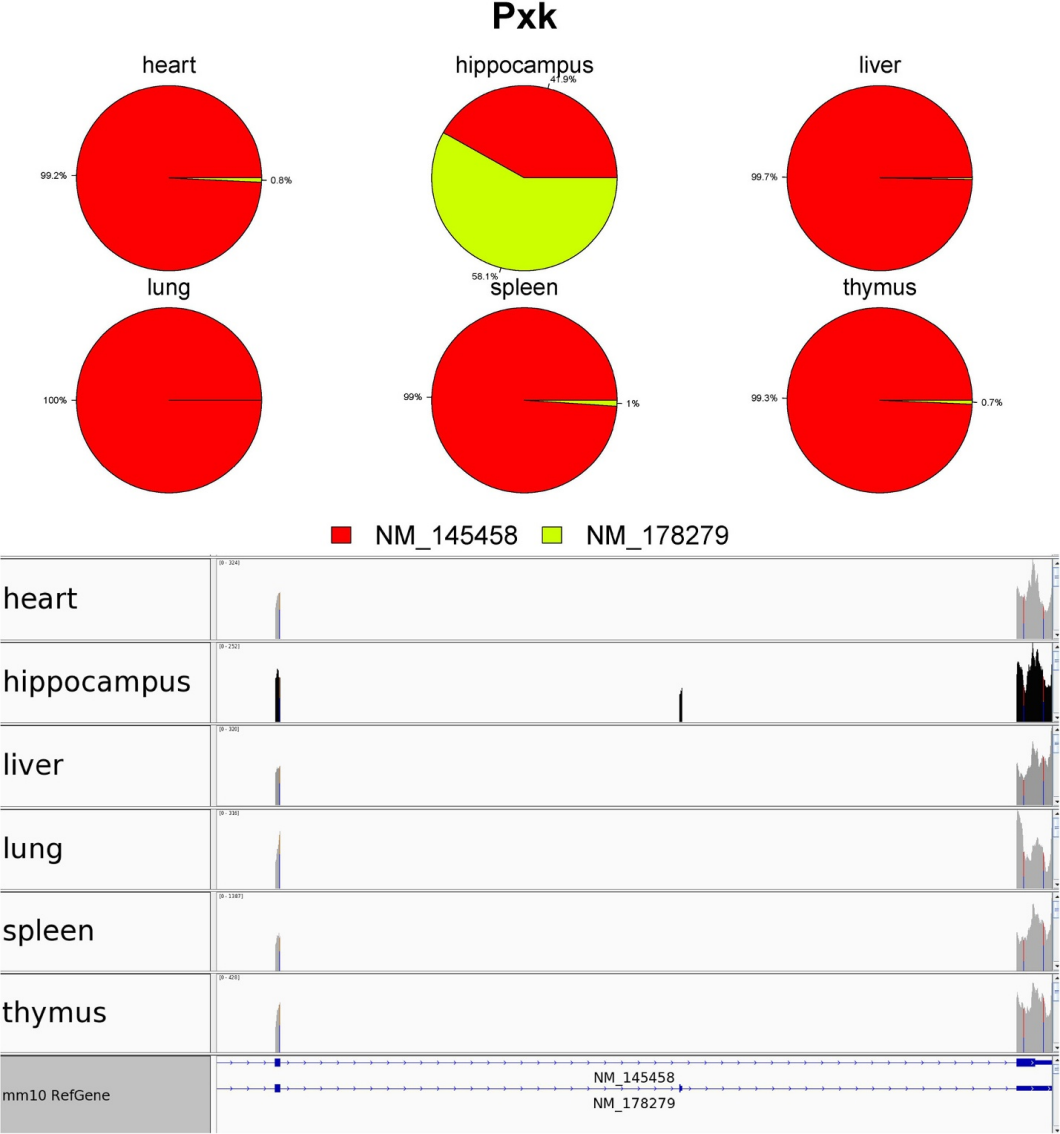
**Visualization of differential isoform usage for** ***Pxk*** **across the six mouse tissues.** (Top) pie plot representations of tissue-specific isoform usage; (bottom) observed RNA-Seq read coverage (in each tissue).

**Figure 3:**
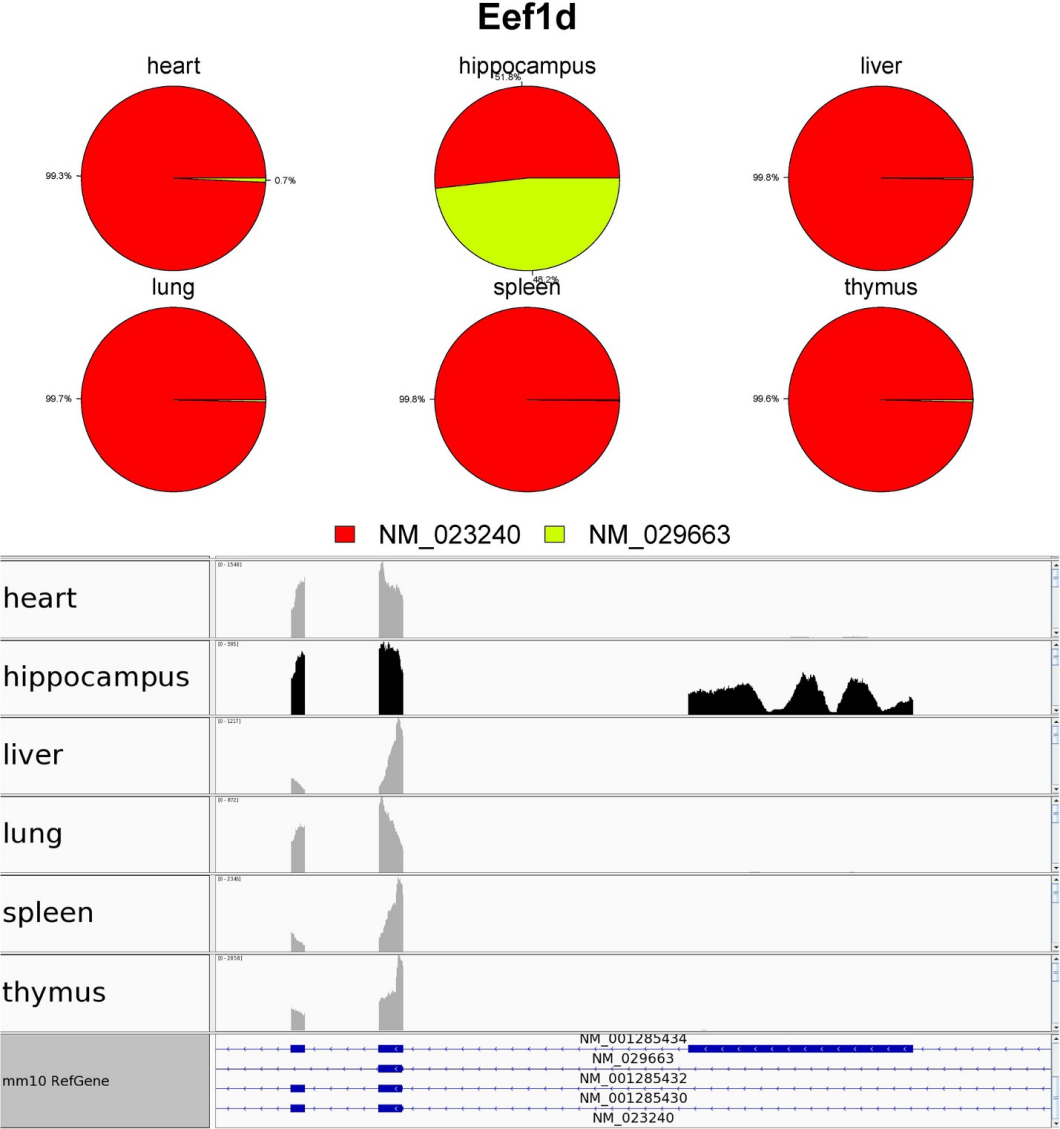
**Visualization of differential isoform usage for** ***Eef1d*** **across the six mouse tissues.** (Top) pie plot representations of tissue-specific isoform usage; (bottom) observed RNA-Seq read coverage (in each tissue).

One such gene is *Pxk*, which has two isoforms (NM_178279 and NM_1454578) (Figure 2). *Pxk*, also known as *MONaKA*, encodes for the PX domain containing serine/threonine kinase. *MONaKA* has been suggested to modulate brain NaK-ATPase and may thereby participate in the regulation of electrical excitability and synaptic transmission [46]. IUTA estimated that NM_178279 was predominantly expressed in hippocampus whereas NM_145458 was predominantly expressed in the other five tissues. Isoform NM_178279 has a unique exon (the middle exon in the Figure 2). Another example is Eef1d (Figure 3), IUTA estimated that NM_029563 has a large relative abundance in hippocampus and has a relative abundance almost 0 in each of the other 5 tissues.

Finally, we applied Cuffdiff2 on the liver and spleen data and compared its performance to that of IUTA. The Cuffdiff2 was also run using the mouse RefSeq gene models. For this data (two groups of six replicates, 27–36 million read pairs in each liver replicate and 8–15 million read pairs in each spleen replicate), both tools run fast, though Cuffdiff2 was faster (8 hours for Cuffdiff2 and 16 hours for IUTA).

**Figure 4:**
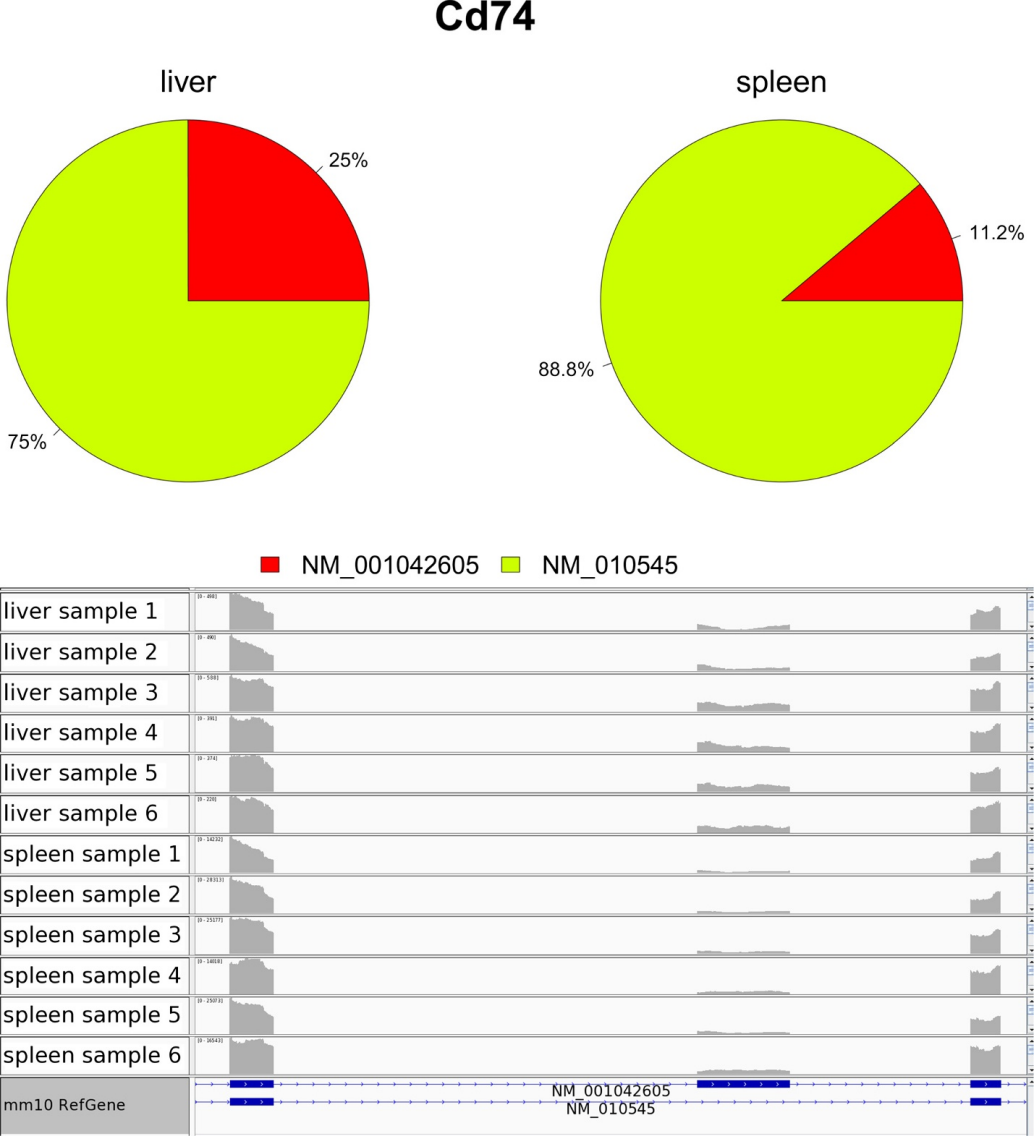
**Visualization of differential isoform usage for** ***Cd74*** **in the mouse liver and spleen tissues.** (Top) pie plot representations of tissue-specific isoform usage; (bottom) observed RNA-Seq reads coverage (in each sample of each tissue).

Among the 2385 mouse genes with more than one isoform and the same transcription start site (in the mouse RefSeq gene model) for all isoforms, IUTA was able to assess statistical significance of isoform usage difference for 1482 genes whereas Cuffdiff2 was able to do 1478 genes given the RNA-Seq data, of which 1268 genes were common between the two tools. IUTA detected more significant genes than Cuffdiff2 did under the same nominal False Discovery Rate (0.05): specifically, Cuffdiff2 reported 122 significant genes whereas IUTA identified 297 significant genes, among which 83 were in common. Visual examination of the isoform usage plots of the genes declared significant by IUTA but not by Cuffdiff2 suggests that the IUTA significant genes are credible. A good example is the *Cd74* gene (Figure 4). Mouse *Cd74* gene has two isoforms. The relative proportion of isoform NM_001042605 is higher in liver than in spleen, as supported by the read coverage plot (Figure 4). The unique (middle) exon in NM_001042605 has higher relative read coverage in liver than in spleen, whereas the read coverage on the common exons appears to be similar among the samples. Although Cuffdiff2 also gave a small p-value (0.020), it failed to reach significance after adjusting for false discovery rate (q-value = 0.14). It is worth pointing out that both IUTA and Cuffdiff2 gave similar isoform usage estimates for this gene. The estimates from IUTA are (0.25, 0.75) in liver and (0.11, 0.89) in spleen, whereas those from Cuffdiff2 are (0.22, 0.78) in liver and (0.11, 0.89) in spleen, respectively. Estimates of isoform usage were often similar between IUTA and Cuffdiff2 even when they differed in declaring a gene to have differential isoform usage between tissues.

## Discussion

Recent advances in next-generation sequencing technologies enable rapid profiling of the entire transcriptome. Methods for identifying differentially expressed genes from RNA-Seq data have been developed, either at the gene level or transcript level. Less attention has been paid, however, to the development of robust and general methods for identifying differential isoform usage (the set of the relative abundances of all isoforms of a gene). Detecting differential isoform usage is different from detecting differential isoform expression, which aims to detect the difference of expression level between two conditions for each individual isoform of each gene. For the latter problem, many excellent tools have been proposed such as MISO [23], Bitseq [22], and EBseq [24]. Among methods for the former problem, Cuffdiff2 has gained recognition as the best available tool. Our proposed method, IUTA, which explicitly makes use of the compositional nature of isoform-usage estimates, offers some advantages compared to the recent completely overhauled version of Cuffdiff2 (http://cufflinks.cbcb.umd.edu). Because MISO, Bitseq, and EBseq are designed for testing a statistically different hypothesis than Cuffdiff2 and IUTA, we did not include the former methods in our simulations.

Using alignments from paired-end reads and a known isoform structure, IUTA first estimates the isoform usage for each gene in each sample from each group and then uses those estimates to test for differential isoform usage between two groups. Because isoform usage is a type of compositional data, i.e., vector of all relative proportions of the whole, Aitchison geometry is more suitable than Euclidean geometry. Aitchison geometry implicitly respects the bounded sample space induced by the isoform usage vector’s summing to one, and its distance metric is fundamentally related to fold-change. Thus, IUTA formulates the problem of testing for differential isoform usage under Aitchison geometry and is, to our knowledge, the first approach to do so.

One aspect of the Aitchison geometry can be problematic, however. Two proportions for the same isoform in different conditions that are both quite small but differ by orders of magnitude (say, 10^−7^ and 10^−10^) sometimes have a strong influence on conclusions. Even though distinguishing such small values is beyond the ability current technology, estimates of isoform usage occasionally contain them. Through the simulation and real data studies, we found that genes with one or more isoforms having near-zero usage in both conditions were often declared to have statistically significant differential usage even when the other entries of the isoform-usage vector were quite similar. To deal with this issue of near-zero estimated usage for some isoforms, IUTA replaces those values, together with sporadic estimated zeros, by a user-specified small positive value (0.0001 by default) to facilitate the testing procedure. Setting an even larger value such as 0.01 would ensure that the top-ranked significant genes are those with a large change in their isoform usages. The user can investigate whether near-zero values are influencing conclusions by seeing whether changes in this tuning parameter impact which genes IUTA declares as having differential isoform usage. This ability represents a practical approach to a knotty problem in compositional data analysis.

A distressing feature of both IUTA and Cuffdiff2 was their failure to maintain the nominal Type I error rate – both rejected the null hypothesis too often when it was true. Because procedures for controlling false discovery rate rely on p values from individual tests, a set of genes declared to have differential isoform usage at a false discovery rate of, say, 0.05 will actually represent a larger, perhaps much larger, false discovery rate when using these two procedures. The most likely reason for the failure of IUTA to control the Type I error rate is that the validity of its tests’ p values is justified theoretically for large numbers of replicate samples but not for the few replicate samples used in studies of isoform usage. We believe that this issue is likely widespread among genome-wide tests that based on only a few replicate samples. We found it true of Cuffdiff2 also. Because many comparisons among competing procedures are based on ROC curves, the failure to properly control Type I error rate or false discovery rate may not be noticed by those developing methods -- as almost happened to us.

An ideal solution to the failure to control Type I error rates in isoform usage testing is elusive. Though much larger numbers of samples in each group would ameliorate the problem, the cost of experiments would be prohibitive. Permutation testing is another option. Although we found that a permutation approach for the tests IUTA_SKK and IUTA_CQ helped to maintain the nominal Type I error rate (see details in the Additional file 1), the minimum possible p values for permutation tests with few samples can be too large to reach statistical significance after FDR or other genome-wide multiple-comparison adjustment. Maintaining the correct Type I error rates will remain a challenge whenever inferences must be based on a relatively small number of replicate samples.

IUTA has several advantages over existing tools: first, IUTA directly incorporates the uncertainty of the underlying DNA fragments corresponding to the paired-end reads into the RNA-Seq data model when estimating the isoform usage. Second, IUTA utilizes all paired-end reads that are mapped to a gene, not only those that are mapped to the unique regions of a gene, to test for significance. Third, IUTA takes into consideration biological variation between by requiring biological replicates and accommodates technical replicates by combining all technical replicates for a given biological replicate into a single data set. Lastly, for each gene, IUTA provides not only the p-value for differential isoform usage for each gene but also the estimate of isoform usage for each gene in each sample.

We evaluated IUTA’s performance through simulation studies in comparison to Cuffdiff2. Cuffdiff2 was designed to test either if alternative TSSs are differentially used (differential promoter usage) between groups of samples or if, for genes with a unique TSS, alternative splicing events occur between two groups of samples. Thus, for testing differential isoform usage, Cuffdiff2 is limited to genes with all isoforms resulting from the same pre-mRNA, i.e., sharing the same TSS. In contrast, IUTA is applicable to all genes with at least two isoforms regardless of their TSS. Consequently, IUTA can test many more genes differential isoform usage than can Cuffdiff2 for the same data. Moreover, we showed that IUTA performed better than Cuffdiff2 for the subset of genes Cuffdiff2 could analyze. IUTA is programmed in R which has the advantage of code that is easily modified and integrated with other tools, such as those available through Bioconductor but has disadvantages in processing speed compared to other languages.

When applied to the tissue transcriptome data from the Mouse Genomes Project from the Wellcome Trust Sanger Institute, IUTA identified 2,073 genes with differential isoform usage between any two given tissues and 46 genes with tissue-specific isoform usage. We believe that our analysis results are the first systematic catalogue of differential isoform usage between the six mouse tissues. We examined a few of those genes and found literature support. Although the functional relevance for many of those genes remains unknown, we believe that the analysis results that we provide in Additional files 2 and 3 for those tissues/cell lines will be useful to those interested in alternative splicing. Of course, all results based on computational tools like IUTA and Cuffdiff2 should be confirmed in the laboratory.

## Conclusions

IUTA, conveniently implemented in an R package, is unique among tools for genome-wide estimation and testing of isoform usage in that it utilizes contemporary statistical techniques for compositional data.

Though the development of computational/statistical methods for genome-wide analysis of alternative splicing is in its early stages, consortiums like TCGA are generating large numbers of RNA-Seq datasets that could be used to probe isoform usage. We expect that new computational methods like ours will catalyse routine genome-wide analysis of alternative splicing, making it as commonplace as genome-wide analysis of gene expression.

## Electronic supplementary material

Additional file 1: **IUTA_Supplementary_Material.** This pdf file contains all supplementary notes and supplementary figures. (DOCX 658 KB)

Additional file 2: **Differential_IU_genes.** This Excel file lists all genes identified by IUTA as exhibiting differential isoform usage between any two of the six mouse tissues. The RNA-Seq data sets were from the Mouse Genomes Project at the Wellcome Trust Sanger Institute (accession number: ERP000591). The file contains 15 columns (one for each of 15 pair-wise comparisons among 6 mouse tissues), the genes identified as having differential isoform usage between two tissues are listed under the column corresponding to those two tissues. The FWER is controlled at 0.01 by Bonferroni correction for each comparison. (XLSX 70 KB)

Additional file 3: **IU_all_mouse_genes.** This Excel file lists the p-values for all the genes that were analyzed by IUTA during the 15 pair-wise comparisons between any two of the six mouse tissues. The RNA-Seq data sets were from the Mouse Genomes Project at the Wellcome Trust Sanger Institute (accession number: ERP000591). The file contains 9 columns (one for the gene symbol, one for the isoforms, six for the estimated isoform usage in the 6 mouse tissues, one for the 15 p-values from the 15 pair-wise differential isoform usage tests). (XLSX 679 KB)
