# Supplementary material for: IUTA: a tool for effectively detecting differential isoform usage from RNA-Seq data
Source: BMC Genomics. 2014 Oct 6;15(1):862. doi: 10.1186/1471-2164-15-862 (PMC4195885; doi:10.1186/1471-2164-15-862)
Supplement: Supplementary file 1 — Additional file 1: IUTA_Supplementary_Material. This pdf file contains all supplementary notes and supplementary figures. (DOCX 658 KB) [file 12864_2014_6529_MOESM1_ESM.docx]

# Supplementary Material For

# IUTA: a tool for effectively detecting differential isoform usage from RNA-Seq data

# Estimating the fragment length distribution

In the likelihood function $L\left( \boldsymbol{\theta} \right)$, the fragment length distribution $f(\cdot)$ is unknown but can be estimated from the alignment data $\boldsymbol{r}$. IUTA estimates $f(\cdot)$ by applying a central moving average filter with a window of length 11 bases to the empirical fragment length distribution determined from each sample. IUTA determines the empirical fragment length distribution by the lengths of the fragments corresponding to the read pairs that are mapped to “stand-alone” exons in the genome, that is, those exons that do not overlap with any other exons. The moving average filter smooths the empirical fragment length distribution. Using simulation studies, we confirmed the utility of mapping to “stand-alone” exons. Specifically, we generated simulated fragments from a discrete normal distribution with mean 250 and standard deviation 10 and compared the mean and the standard deviation of the empirical fragment length distribution determined by IUTA with the true values. Regardless of the average read coverage and the number of genes used in the simulation, the absolute difference between the estimated mean and true mean was always less than 0.9 and the absolute difference between the estimated standard deviation and true standard deviation was always less than 0.1.

# EM algorithm to find the Maximum Likelihood estimate (MLE) of $\boldsymbol{\theta}$

To find the MLE of isoform usage vector $\boldsymbol{\theta=}(\theta_{1}, \cdots, \theta_{K})$**,** denoted by $\hat{\boldsymbol{\theta}}=(\hat{\theta_{1}}, \cdots, \hat{\theta_{K}})$, we first use an Expectation-Maximization (EM) algorithm to find the MLE of $\boldsymbol{p=}(p_{1}, \cdots, p_{K})$ ($p_{k}$ is the probability of observing a paired-end read from isoform $k$, where $1\leq k\leq K$), denoted by $\hat{\boldsymbol{p}}=(\hat{p_{1}},\cdots,\hat{p_{K}})$. Recall that $p_{k}= \frac{l_{k}\theta_{k}}{\sum_{u=1}^{K} l_{u}\theta_{u}}$ where $l_{k}$ is the length of isoform $k$. Then $\hat{\boldsymbol{\theta}}$ is calculated from the EM-estimate $\hat{\boldsymbol{p}}$ using $\hat{\theta_{k}}= \frac{\hat{p_{k}}{/l}_{k}}{\sum_{u=1}^{K} \hat{p_{u}}/l_{u}}$ ($1\leq k\leq K$).

The E-step and M-step of the EM algorithm for finding $\hat{\boldsymbol{p}}$ are as follows. This algorithm treats each $I_{n}$, i.e., the isoform from which alignment $r_{n}$ is generated, as an unobserved latent variable.

The E step involves calculating the expected value of log-likelihood function with respect to the conditional distribution of $\boldsymbol{I}=(I_{1},\cdots, I_{N})$ given $\boldsymbol{R}=\boldsymbol{r=}(r_{1},\cdots, r_{N})$ under the current estimate at iteration *t*, namely, ${\hat{\boldsymbol{p}}}^{t}=\left( {\hat{p_{1}}}^{t}, \cdots, {\hat{p_{K}}}^{t} \right)$. That is, one calculates

$$E_{\boldsymbol{I}|\boldsymbol{R},{\hat{\boldsymbol{p}}}^{t}}\left( \log L\left( \boldsymbol{p} \right) \right)=E_{\boldsymbol{I}|\boldsymbol{R},{\hat{\boldsymbol{p}}}^{t}}\left( \log P\left( \boldsymbol{R},\boldsymbol{I} | \boldsymbol{p} \right) \right)$$

$$=\sum_{n=1}^{N} E_{\boldsymbol{I}|\boldsymbol{R},{\hat{\boldsymbol{p}}}^{t}}\left( \log P\left( R_{n}=r_{n},I_{n} | \boldsymbol{p} \right) \right).$$

Because knowing that $r_{n}$ is from isoform $k$ determines $l_{k}^{n}$ (the length of the fragment of isoform $k$ that matches $r_{n}$), $P\left( R_{n}=r_{n},I_{n}=k | \boldsymbol{p} \right)=P\left( R_{n}=r_{n},L_{n}=l_{k}^{n},I_{n}=k | \boldsymbol{p} \right)$ where $L_{n}$ is the random variable that represent the length of the fragment from which $r_{n}$ is sequenced. The right-hand side of this equality can be factored as:

$$P\left( I_{n}=k | \boldsymbol{p} \right)P\left( L_{n}=l_{k}^{n} \right|I_{n}=k,\boldsymbol{p})P\left( R_{n}=r_{n} \right|L_{n}=l_{k}^{n},I_{n}=k,\boldsymbol{p}).$$

Substituting the notation established in the manuscript into the preceding expression yields:

$$P\left( R_{n}=r_{n},I_{n} | \boldsymbol{p} \right)=p_{k}f\left( l_{k}^{n} \right)\frac{1}{l_{k}-l_{k}^{n}+1}.$$

Using this result in the calculation of $E_{\boldsymbol{I}|\boldsymbol{R},{\hat{\boldsymbol{p}}}^{t}}\left( \log P\left( R_{n}=r_{n},I_{n} | \boldsymbol{p} \right) \right)$ yields the following expression:

$$E_{\boldsymbol{I}|\boldsymbol{R},{\hat{\boldsymbol{p}}}^{t}}\left( \log L\left( \boldsymbol{p} \right) \right)=\sum_{n=1}^{N} \sum_{k=1}^{K} [\log\left( p_{k} \right)+log(f\left( l_{k}^{n} \right)\frac{1}{l_{k}-l_{k}^{n}+1})]\cdot P(I_{n}=k|r_{n},{\hat{\boldsymbol{p}}}^{t})$$

$$= \sum_{n=1}^{N} \sum_{k=1}^{K} \log\left( p_{k} \right)\cdot P\left( I_{n}=k | r_{n},{\hat{\boldsymbol{p}}}^{t} \right)+C,$$

where

$$C=\sum_{n=1}^{N} \sum_{k=1}^{K} log(f\left( l_{k}^{n} \right)\frac{1}{l_{k}-l_{k}^{n}+1})\cdot P(I_{n}=k|r_{n},{\hat{\boldsymbol{p}}}^{t})$$

is a constant that does not depend on $\boldsymbol{p}$ because neither $l_{k}^{n}$ nor $P(I_{n}=k|r_{n},{\hat{\boldsymbol{p}}}^{t})$ depend on $\boldsymbol{p}$.

The M step involves maximizing $E_{\boldsymbol{I}|\boldsymbol{R},{\hat{\boldsymbol{p}}}^{t}}\left( \log P\left( \boldsymbol{R},\boldsymbol{I} | \boldsymbol{p} \right) \right)$ under the constraint $\sum_{k=1}^{K} p_{k}=1$ to update ${\hat{\boldsymbol{p}}}^{t}$ to ${\hat{\boldsymbol{p}}}^{t+1}$. This maximization uses the Lagrange multiplier technique; one solves the following equation system, where $\lambda$ is the Lagrange multiplier:

$$\left\{ \begin{aligned} \sum_{n=1}^{N} \frac{P(I_{n}=1|r_{n},{\hat{\boldsymbol{p}}}^{t})}{p_{1}}+\lambda=0 \\ \cdots\\ \sum_{n=1}^{N} \frac{P(I_{n}=K|r_{n},{\hat{\boldsymbol{p}}}^{t})}{p_{K}}+\lambda=0 \\ \sum_{k=1}^{K} p_{k}=1 \end{aligned} \right.\text{.}$$

The solution is ${\hat{\boldsymbol{p}}}^{t+1}=({\hat{p_{1}}}^{t+1}, \cdots, {\hat{p_{K}}}^{t+1})$ with

$${\hat{p_{k}}}^{t+1}=\frac{\sum_{n=1}^{N} P\left( I_{n}=k | r_{n}, {\hat{\boldsymbol{p}}}^{t} \right)}{N}\text{,}$$

where $p\left( I_{n}=k | r_{n},{\hat{\boldsymbol{p}}}^{t} \right)=\frac{{\hat{p_{k}}}^{t}c_{k}^{n}}{\sum_{u=1}^{K} {\hat{p_{u}}}^{t}c_{u}^{n}}$ with $c_{k}^{n}=f\left( l_{k}^{n} \right)\frac{1}{l_{k}-l_{k}^{n}+1}$ ($1\leq k\leq K$).

IUTA calculates starting values for $\boldsymbol{p}$ in the EM algorithm as follows. First, for each $r_{n}$ ($1\leq n\leq N$), IUTA calculates $f\left( l_{k}^{n} \right)$ for $1\leq k\leq K$, counts the number of non-zero values of $f(l_{k}^{n})$, say $c_{n}$, and assigns $1/c_{n}$ to the $c_{n}$ isoforms where $f(l_{k}^{n})$ is non-zero and assigns 0 to the remaining isoforms, forming a $K$-dimensional probability vector. For example, suppose that with five isoforms ($K=5$) and a given aligned read, the corresponding values of $f\left( l_{k}^{n} \right)$ were 0, 0.01, 0.01, 0 and 0.02 for the five isoforms, respectively. IUTA would assign the 5-dimensional probability vector $\left( 0,\frac{1}{3},\frac{1}{3},0,\frac{1}{3} \right)$ to isoforms 1 through 5, respectively, as the probability that the given read came from each of the isoforms. IUTA performs the same calculation for every aligned read, and sums all the resulting $K$-vectors. After re-scaling so the elements in the summed vector add to 1, IUTA uses the vector as the starting value for $\boldsymbol{p}$. Note that IUTA removes any reads that are not consistent with any isoform of the gene.

# A brief explanation of Aitchison geometry

The isoform usage data is a type of compositional data [[1](#_ENREF_1)], i.e., proportions of a whole. Because the sum of the proportions is one (100%), the sample space is for compositional data is a bounded space (known as a simplex), Euclidean geometry is unsuitable [[2](#_ENREF_2)]. A commonly accepted geometry for compositional data analysis is the Aitchison geometry [[3](#_ENREF_3)], which, in effect, deals with log ratios of the proportions. One common approach to making statistical inference on compositional data in Aitchison geometry is to use an isometric log-ratio (ilr) transformation [[4](#_ENREF_4)]. This kind of transformation is a distance-preserving one-to-one mapping between $\mathcal{S}^{K}$ (the open simplex with Aitchison geometry) and $\mathbb{R}^{K-1}$(the real space with Euclidean geometry). It transforms a $K$-dimensional compositional vector to a $(K-1)$-dimensional Euclidean vector so that familiar inference techniques can be applied to the transformed data. Accordingly, the most widely used random distribution for a vector in $\mathcal{S}^{K}$ is the so-called normal distribution on the open simplex $\mathcal{S}^{K}$, which corresponds to the normal distribution on $\mathbb{R}^{K-1}$ for the ilr-transformed random composition variable. IUTA assumes that the isoform usage in each sample follows a group-specific normal distribution on the open simplex. The test for differential isoform usage, after transformation, becomes a test of whether the means of the two normal distributions are equal.

The mean of a random variable in Aitchison geometry can be understood as follows. Because an ilr transformation exists between $\mathcal{S}^{K}$ and $\mathbb{R}^{K-1}$, the law of large numbers that holds in $\mathbb{R}^{K-1}$ also holds in $\mathcal{S}^{K}$. Consequently, the mean of a random variable in $\mathcal{S}^{K}$ can be viewed as the value to which the sample average converges almost surely (in Aitchison geometry). Consider a set of $N$ points $\{\boldsymbol{x}_{n}: 1\leq n\leq N\}$ in $\mathcal{S}^{K}$, where $\boldsymbol{x}_{n}\boldsymbol{=(}x_{1n}, \cdots, x_{Kn})$. In Aitchison geometry, the average of $\{\boldsymbol{x}_{n}: 1\leq n\leq N\}$ is $\boldsymbol{m=(}m_{1}, \cdots, m_{K})$, where $m_{k}=d\times\left( \prod_{n=1}^{N} x_{kn} \right)^{\frac{1}{N}}$ for $1\leq k\leq K$ and $d$ is a constant chosen so that $\sum_{k=1}^{K} m_{k}=1$. In Aitchison geometry, the distance between two points $\boldsymbol{x=(}x_{1}\boldsymbol{,}\cdots, x_{K}\boldsymbol{)}$ and $\boldsymbol{y}=(y_{1},\cdots, y_{k})$ is $\sqrt{\frac{1}{2k}\sum_{k=1}^{K} \sum_{l=1}^{K} \left( log(\frac{x_{k}}{x_{l}})-log(\frac{y_{k}}{y_{l}}) \right)^{2}}$.

An ilr transformation is not unique. The particular one that IUTA uses is defined as follows. For $\boldsymbol{x=(}x_{1}\boldsymbol{,}\cdots, x_{K}\boldsymbol{)}$ in $\mathcal{S}^{K}$, $\mathrm{ilr}\left( \mathbf{x} \right)=\log\left( \mathbf{x} \right)\times\boldsymbol{\Psi}$, where $\log\left( \mathbf{x} \right)=(\log\left( x_{1} \right), \cdots, log(x_{K}))$ (viewed as a $1\times K$ matrix) and $\boldsymbol{\Psi=(}\Psi_{\mathrm{ij}}\mathbf{)}$ is a $K\times(K-1)$ matrix with elements

$$\Psi_{\mathrm{ij}}=\left\{ \begin{aligned} \frac{1}{\sqrt{\left( K-j \right)\left( K-j+1 \right)}}, if i\leq K-j \\ -\frac{\sqrt{K-j}}{\sqrt{K-j+1}}, if i=K-j+1 \\ 0, else \end{aligned} \right..$$

For example, when $K=5$, $\boldsymbol{\Psi}\mathbf{=}\left[ \begin{matrix} \frac{1}{\sqrt{20}} & \frac{1}{\sqrt{12}} & \frac{1}{\sqrt{6}} & \frac{1}{\sqrt{2}} \\ \frac{1}{\sqrt{20}} & \frac{1}{\sqrt{12}} & \frac{1}{\sqrt{6}} & -\frac{1}{\sqrt{2}} \\ \frac{1}{\sqrt{20}} & \frac{1}{\sqrt{12}} & -\frac{\sqrt{2}}{\sqrt{3}} & 0 \\ \frac{1}{\sqrt{20}} & -\frac{\sqrt{3}}{\sqrt{4}} & 0 & 0 \\ -\frac{2}{\sqrt{5}} & 0 & 0 & 0 \end{matrix} \right]$**.**

Notice that the $j$-th column in $\boldsymbol{\Psi}$ is a vector, standardized to length 1, that compares the average of the first $\left( K-j \right)$ components of $\log\left( \mathbf{x} \right)$ to the $\left( K-j+1 \right)$ component.

# Two sample test for multivariate normal distributions with unequal variance-covariance matrices

To test $H_{0}'$ versus $H_{1}'$ using the values of ${ilr(\hat{\boldsymbol{\theta}}}_{ij})$, we are in effect testing if the means of two multivariate normal distributions are equal while allowing their variance-covariance matrices to be unequal. This testing problem is known as the *Behrens-Fisher* problem. For the univariate case, Welch’s $t$-test [[5](#_ENREF_5)] is typically used. For larger values of $K$, no approach is commonly accepted yet, although many methods have been proposed since 1940’s. Among those methods, the test proposed in [[6](#_ENREF_6)], called the *KY test* in this paper, is a generalization of the Welch’s test and is recommended by [[7](#_ENREF_7)]. However, KY test cannot be applied when $\left( K-1 \right)\geq min(J_{0}, J_{1})$, where $J_{0}$ and $J_{1}$are the number of samples for two groups – that is, when either estimated variance-covariance matrix is singular (not positive definite). In practice, $J_{0}$ and $J_{1}$ are usually between 2 to 5 and *K* is at least 5. For this reason, we adopt two additional tests that can accommodate singular variance-covariance matrices: the SKK test proposed in [[8](#_ENREF_8)] and the CQ test proposed in [[9](#_ENREF_9)]. These two tests employ different test statistics: the SKK test is invariant under the units of measurements while CQ test is not[[8](#_ENREF_8)]. The SKK test can outperform the CQ test [[8](#_ENREF_8)]. All three tests are implemented in the R package of IUTA and are sometimes referred to as IUTA_SKK, IUTA_CQ and IUTA_KY in this paper. From simulation studies, we found based on ROC curves that the SKK test and the CQ test outperformed the KY test when the KY test is applicable (i.e., when the estimated group-specific variance-covariance matrices were positive definite) and that the SKK test performed comparably with the CQ test when $(K-1)$ was no less than the number of samples. IUTA uses the SKK test as its default.

# Simulated data generation

We performed three simulation studies for different purposes. The first one aimed to compare the three tests implemented in IUTA (SKK, CQ and KY) and to compare IUTA (with SKK) with Cuffdiff2 (version 2.2.0). The second simulation study probed the robustness of IUTA to violation of the constant variance-covariance assumption that $\boldsymbol{\Upsilon}_{ij}=\boldsymbol{\Upsilon}_{i}$ for$1\leq j\leq J_{i}$. The third aimed to assess the robustness of IUTA to variation in read coverage among samples.

In the first two simulation studies, we selected 8,628 mouse genes with at least two isoforms but no more than 10 (see gene selection below). We divided the 8,628 genes into two subsets (8,060 genes with 2-5 isoforms and 568 with 6-10 isoforms) and each subset was investigated separately. Each of these two simulation studies consisted of one *in silico* experiment for each subset of genes. A single experiment involved 10 randomly generated alignment BAM data sets for the appropriate subset of genes; the 10 data sets represented 5 samples from each of two groups. For the first simulation study, each gene in all samples had the average read coverage set at 100. In the second simulation study, the average read coverage differed across the five samples from each group: read coverage was set at 30, 50, 70, 90, and110 for the 5 samples, respectively.

In the third simulation study, we randomly selected five genes (*Zfp407*, *Loxl2*, *Bptf*, *Pde4dip,* and *Stab2*) with 2, 3, 5, 7, and 8 isoforms, respectively; we also selected another two genes (*Ddo1* and *Ifi203*), each with 8 isoforms. For each of these seven genes, we studied six different average read coverages (10, 30, 50, 70, 90, and 110). For each read coverage and each gene, we simulated 1,000 independent replicate *in silico* experiments consisting of 10 data sets comprising 5 samples from each of two groups, as before.

## **Selection of genes**

We downloaded the UCSC known gene annotation GTF file from the UCSC genome browser. For our analyses, we eliminated genes with the following characteristics: a) with only one isoform; b) located on “non-standard” chromosomes such as (“chrN_*_random”, “chrUn”, “chrM”); c) located on multiple chromosomes; d) with isoforms in different orientations (+ and -); d) with short isoforms (< 200 base pairs); e) with more than 10 isoforms. The reason for removing genes with more than 10 isoforms is that, with only had 10 RNA-Seq datasets from our collaborators, the estimated variance-covariance matrices for genes with more than 10 isoforms would be singular, and we wanted to avoid using singular matrices as simulation parameters.

## **Determination of the simulation parameters**

We based the simulation parameters for each of the selected 8628 genes, including $\boldsymbol{\Sigma}_{0}$ and $\boldsymbol{\Sigma}_{1}$ of Equation (1) and the distance between $\boldsymbol{\theta}_{0}$ and $\boldsymbol{\theta}_{1}$ under the alternative hypothesis, on 10 mouse placenta RNA-Seq data sets (two groups, five wild-type and five *Zfp36l3* knockout) (unpublished) provided by Perry Blackshear (NIEHS).

To determine $\boldsymbol{\Sigma}_{0}$ and $\boldsymbol{\Sigma}_{1}$ of Equation (1), we first ran Tophat [[10](#_ENREF_10)] on the each of the 10 data sets to map the reads to the mouse genome (mm10) according to the UCSC known gene annotation and then ran Cufflinks [[11](#_ENREF_11)] on the resulting alignment BAM file to obtain the initial estimates of the isoform abundances (in units of FPKM, i.e., Fragments Per Kilobase of exon per Million fragments mapped) for each gene. We then used those estimates to estimate the isoform usage for each gene in each sample, and determined the $\boldsymbol{\Sigma}_{0}$ and $\boldsymbol{\Sigma}_{1}$ for each gene using the total 10 ilr-transformed estimated isoform usages. Specifically, for each gene with 2-5 isoforms (8,060 genes), we calculated the sample variance-covariance matrix using the five ilr-transformed isoform usage estimates in each group. Notice that this estimation procedure actually estimates $(\boldsymbol{\Sigma}_{0}\boldsymbol{+}\boldsymbol{\Upsilon}_{0})$ and $(\boldsymbol{\Sigma}_{1}\boldsymbol{+}\boldsymbol{\Upsilon}_{1})$ for each gene, but we used those estimates as realistic values for setting the values of $\boldsymbol{\Sigma}_{0}$ and $\boldsymbol{\Sigma}_{1}$, respectively, in our simulations. For each gene with 6-10 isoforms (568 genes), we calculated one sample variance-covariance matrix using all 10 ilr-transformed isoform usage estimates. In the simulations for each gene, we used the single estimated variance-covariance matrix for that gene as both $\boldsymbol{\Sigma}_{0}$ and $\boldsymbol{\Sigma}_{1}$.

To set the distance between $\boldsymbol{\theta}_{0}$ and $\boldsymbol{\theta}_{1}$ for each gene under the alternative hypothesis, we averaged (in Aitchison geometry) the estimated isoform usage of the five samples in each group and computed the distance in Aitchison geometry between the two averages for all the 8628 selected genes. In each simulation, the distance between $\boldsymbol{\theta}_{0}$ and $\boldsymbol{\theta}_{1}$ under the alternative hypothesis for a gene was then sampled uniformly from the top 5% of such distances in the subset of genes to which the gene belonged (either the set of 8060 genes or the set of 568 genes).

## **Simulation procedures**

In each *in silico* experiment, we followed the following steps gene by gene to get the 10 simulated alignment BAM data sets. First, we set the probability that any gene had differential isoform usage to be 0*.*2, that is, with a chance of 20% we set the gene to have differential isoform usage between the two groups, and otherwise we set the gene to have identical isoform usage between the two groups. Second, we sampled a value uniformly on the open simplex $\mathcal{S}^{K}$, where $K$ is the number of isoforms of the gene, and used the value as the mean isoform usage for the gene in group 0, i.e., the $\boldsymbol{\theta}_{0}$ in Equation (1). To generate such a random sample on $\mathcal{S}^{K}$, we generated $K-1$ locations uniformly distributed on the interval $(0, 1)$; the lengths of the $K$ subintervals formed by the union these $K-1$ locations and the endpoints 0 and 1 is a $K$-dimensional vector on $\mathcal{S}^{K}$. Third, under the alternative hypothesis, we randomly chose a value $d$ from the top 5% of the Aitchison distances obtained as described above, sampled a value uniformly on the sphere in Aitchison geometry centered at $\boldsymbol{\theta}_{0}$ with radius $d$, and designated the sampled value as the mean isoform usage under group 1 ($\boldsymbol{\theta}_{1}$). To sample a value uniformly on the sphere centered at $\boldsymbol{\theta}_{0}$ with radius $d$, we took a sample in $\mathbb{R}^{K-1}$ from the uniform distribution on the sphere centered at ${ilr(\boldsymbol{\theta}}_{0})$ with radius $d$ and then back transformed the sample by applying $ilr^{-1}$, the inverse of the ilr transformation. We sampled from the uniform distribution on the sphere of radius $d$ centered at ${ilr(\boldsymbol{\theta}}_{0})$ by taking $K-1$ samples from a standard normal distribution, scaling the resulted $(K-1)$-dimensional vector so that it had length $d$, and taking the sum of ${ilr(\boldsymbol{\theta}}_{0})$ and this standardized vector. Fourth, we used the $\boldsymbol{\theta}_{0}$ and $\boldsymbol{\theta}_{1}$, together with $\boldsymbol{\Sigma}_{0}$ and the $\boldsymbol{\Sigma}_{1}$, to simulate $\boldsymbol{\theta}_{ij}$’s, where $i=0, 1$and $1\leq j\leq5$. Finally, we generated the alignments using the simulated $\boldsymbol{\theta}_{ij}$ for sample $j$ of group $i$. Specifically, we first repeated a process (described later) to generate $\frac{c\cdot L}{200}$ DNA fragments using the gene’s isoforms, where $L$ is the length of the union of all the exons of the gene and $c$ is the desired coverage for the gene in the sample. Note that by generating $\frac{c\cdot L}{200}$ fragments, we control the average read coverage to equal the nominal coverage $c$. Next, we took the two 100 base pair genomic regions from the two ends of each fragment and shifted them (independently) with a probability 0.005 along the genome. The details of the shifting are described later. Lastly, we recorded the locations of the (possibly shifted) paired genomic regions as the alignment data for the gene.

We sampled a DNA fragment for a gene as follows. First, we sampled an isoform of the gene according to $\boldsymbol{\theta}_{ij}$, its isoform usage in the sample. Second, we split the sampled isoform into fragments using a Poisson process with mean 250 (base pairs). Third, we sampled the resulting fragments according to their lengths approximated by a discrete normal distribution [[12](#_ENREF_12)] with mean of 250 and standard deviation of 10, i.e., a fragment with length $l$ is selected with probability $P\left( l \right)=\Phi\left( \frac{l+1-250}{10} \right)-\Phi\left( \frac{l-250}{10} \right)$, where $\Phi$ is the cdf of the standard normal distribution. We chose the mean (250) and the standard deviation (10) to simulate a typical RNA-Seq experiment.

A fragment was shifted or not at random. The length of the target fragment was first sampled from the above discrete normal distribution. If only one end was to be shifted (each end independently with probability $0.005\times0.995$), we just moved the 100 bp of the corresponding end by the difference between the target length and the original length (sign of the difference determines the direction of the shift). If both ends were to be shifted (with probability ${0.005}^{2}$), we then randomly selected a starting position on the isoform from which the fragment was obtained, and moved the left end of the original fragment to the chosen position, retaining the original length; then, we either shifted the new left end or the new right end (with equal chance) by the difference between the target length and the original length (sign of the difference determined the direction of the shift).

# Simulation results

## Comparisons of the three IUTA tests and comparison of IUTA with Cuffdiff2

Based on the first simulation study, the ROC curves plot the false positive rate (the proportion of true negatives that are claimed as positives) versus the true positive rate (the proportion of true positives that are claimed as positives) as the p-value cut-offs vary. The SKK test and CQ test performed comparably and they outperformed the KY test for the genes with 2-5 isoforms (**Figure S1**). The SKK test performed comparably to the CQ test for genes with 6-10 isoforms. Note that the KY test was not applicable to genes with 6-10 isoforms as it requires more samples per group than the number of isoforms minus one.

Cuffdiff2 was applicable to only 4159 of the 8628 genes. For those genes, IUTA outperformed Cuffdiff2 (**Figure S1**). There are two reasons why Cuffdiff2 was only applicable to the 4159 genes: 1) Cuffdiff2 is not specifically designed to test for the overall differential isoform usage, but rather to test for a differential splicing event from a single transcription start site (TSS) (4247 genes had a single TSS); 2) when a gene has a single TSS but several isoforms are too similar, Cuffdiff2 cannot provide valid tests, reporting ”NOTEST” due to “no enough alignments for testing”.

Figure S1: Performance comparisons among the three tests (SKK, CQ, and KY) and between IUTA and Cuffdiff2 as shown in Receiver Operating Characteristic (ROC) curves. (a): comparison among the three tests (IUTA_KY, IUTA_SKK, and IUTA_CQ) on 8060-30=8030 genes with 2-5 isoforms (there were 30 genes for which KY test was not applicable due to computing issues). (b): comparison among the two tests (IUTA_SKK and IUTA_CQ) on 568 genes with 6-10 isoforms. (c): comparison between IUTA (IUTA_SKK) and Cuffdiff2 on 4159 genes.

## Robustness to violations of the assumption that $\boldsymbol{\Upsilon}_{\boldsymbol{ij}}\mathbf{=}\boldsymbol{\Upsilon}_{\boldsymbol{i}}$ for$\boldsymbol{1\leq}\boldsymbol{j}\boldsymbol{\leq}\boldsymbol{J}_{\boldsymbol{i}}$.

Based on the second simulation study, as in the first, all three tests implemented in IUTA performed similarly (**Figure S2**). The SKK test performed comparably to the CQ test for genes with 6-10 isoforms. Based on the limited number of genes (4136 out of 8628 genes) that can be tested by Cuffdiff2, IUTA with the SKK test outperformed Cuffdiff2. In this simulation, Cuffdiff2 only analyzed 4136 genes because the variable coverage increased the number of genes which Cuffdiff2 declared as having too few alignments for a valid test.

Figure S2: Performance comparisons among the three tests (SKK, CQ, and KY) and between IUTA and Cuffdiff2 as shown in Receiver Operating Characteristic (ROC) curves, when the constant variance-covariance assumption in equation (1) is violated by differences in read coverage among the samples in each group (either 30, 50, 70, 90, or 110). (a): comparison among the three tests (IUTA_KY, IUTA_SKK, and IUTA_CQ) on 8060-26=8034 genes with 2-5 isoforms (there were 26 genes for which KY test was not applicable due to computing issues). (b): comparison among the two tests (IUTA_SKK and IUTA_CQ) on 568 genes with 6-10 isoforms. (c): comparison between IUTA (IUTA_SKK) and Cuffdiff2 on 4136 genes.

# Maintain nominal type I error rate by a permutation approach

## Via simulations, we found that, although IUTA_KY approximately maintained the nominal Type I error rate, IUTA_SKK, IUTA_CQ and Cuffdiff2 did not, that is, they rejected the null hypothesis too often when it is true. The p-values for the latter three tests, but not IUTA_KY, rely on the validity of large-sample approximations, which are problematic for the small number of replicates typical for RNA-seq experiments. Consequently, we investigated whether a permutation approach might improve control of Type I error rate for IUTA_SKK and IUTA_CQ. Specifically, in the simulation study that used five samples in each group, after we obtained the p-value using IUTA_SKK or IUTA_CQ for a gene, we then permuted the group labels on the estimated isoform usages and performed the test again based on the new group labels. After we ran over all possible permutations and calculated a p-value for each one, we then used the proportion of p-values that were less than or equal to the original p-value to be the new p-value for the gene. Using permutation-based p-values allowed IUTA_SKK and IUTA_CQ to better maintain the nominal Type I error rate in the simulations (Figure S3). Also, the advantages of IUTA_SKK and IUTA_CQ over IUTA_KY in ROC performance persisted under permutation testing (Figure S4).

##

Figure S3: The achieved type I error rate (y-axis) versus the nominal type I error rate (x-axis) for IUTA tests in the simulation study, the curves for IUTA_SKK and IUTA_CQ are based on permutation-adjusted p-values while that for IUTA_KY is based on the original p-values.

Figure S4: Performance comparison among the five tests (SKK with permutation adjustment, CQ with permutation adjustment, SKK, CQ and KY) in the simulation study as shown in Receiver Operating Characteristic (ROC) curves. The ROC curve for IUTA_KY is based on 8030 genes with 2-5 isoforms and the other ROC curves are based on all 8628 genes with 2-10 isoforms.

Permutation testing, though offering improvements, is not a panacea here, however. The smallest p value that can be calculated by permutations depends on the sample size in each group. For example, with only three samples in each group, even if the observed configuration represents the most extreme difference between the two groups, the p-value will not reach 0.05. Even for larger numbers of replicates, the minimal p-value from permutations may not be sufficiently small to allow for stringent multiple testing corrections.

**Supplementary references**

1. Aitchison J: **The statistical analysis of compositional data**: Chapman & Hall, Ltd.; 1986.

2. Pearson K: **Mathematical Contributions to the Theory of Evolution.--On a Form of Spurious Correlation Which May Arise When Indices Are Used in the Measurement of Organs**. *Proceedings of the Royal Society of London* 1896, **60**(359-367):489-498.

3. Pawlowsky-Glahn V, Egozcue JJ: **Geometric approach to statistical analysis on the simplex**. *Stochastic Environmental Research and Risk Assessment* 2001, **15**(5):384-398.

4. Egozcue JJ, Pawlowsky-Glahn V, Mateu-Figueras G, Barceló-Vidal C: **Isometric logratio transformations for compositional data analysis**. *Mathematical Geology* 2003, **35**(3):279-300.

5. Welch BL: **The generalization ofstudent's' problem when several different population variances are involved**. *Biometrika* 1947, **34**(1/2):28-35.

6. Krishnamoorthy K, Yu J: **Modified Nel and Van der Merwe test for the multivariate Behrens–Fisher problem**. *Statistics & probability letters* 2004, **66**(2):161-169.

7. Zezula I: **Implementation of a new solution to the multivariate Behrens-Fisher problem**. *Stata Journal* 2009, **9**(4):593-598.

8. Srivastava MS, Katayama S, Kano Y: **A two sample test in high dimensional data**. *Journal of Multivariate Analysis* 2013, **114**:349-358.

9. Chen SX, Qin Y-L: **A two-sample test for high-dimensional data with applications to gene-set testing**. *The Annals of Statistics* 2010, **38**(2):808-835.

10. Kim D, Pertea G, Trapnell C, Pimentel H, Kelley R, Salzberg SL: **TopHat2: accurate alignment of transcriptomes in the presence of insertions, deletions and gene fusions**. *Genome Biol* 2013, **14**(4):R36.

11. Trapnell C, Williams BA, Pertea G, Mortazavi A, Kwan G, van Baren MJ, Salzberg SL, Wold BJ, Pachter L: **Transcript assembly and quantification by RNA-Seq reveals unannotated transcripts and isoform switching during cell differentiation**. *Nat Biotechnol* 2010, **28**(5):511-515.

12. Roy D: **The discrete normal distribution**. *Communications in Statistics-Theory and Methods* 2003, **32**(10):1871-1883.
